# High impact of chemotherapy on ovarian reserve in breast cancer survivors of reproductive age: A systematic review and meta-analysis

**DOI:** 10.1016/j.breast.2025.104514

**Published:** 2025-06-13

**Authors:** Susanna Weidlinger, Magdalena Weidlinger, Rose-Maria Schramm, Angela Vidal, Janna Pape, Tanya Karrer, Manuela Rabaglio, Michael von Wolff

**Affiliations:** a Division of Gynecological Endocrinology and Reproductive Medicine, University Women's Hospital, Inselspital Bern, University of Bern, 3010, Bern, Switzerland; b Medical Library, University Library Bern, University of Bern, 3010, Bern, Switzerland; c University Clinic for Medical Oncology, Inselspital Bern, University of Bern, 3010, Bern, Switzerland

**Keywords:** Anti-Mullerian hormone, AMH, Chemotherapy, Breast cancer, Ovarian reserve, Fertility, FertiTOX

## Abstract

**Introduction:**

The risk of infertility following breast cancer (BC) treatment is critical for women of reproductive age. Accurate risk assessment is essential for fertility counseling and preservation. Amenorrhoea as an infertility marker is unreliable due to endocrine therapies. Anti-Mullerian hormone (AMH) is a reliable fertility marker, but its role in assessing chemotherapy-induced loss of ovarian reserve in BC survivors remains underexplored.

**Objective:**

This systematic review and meta-analysis evaluates AMH decline and the prevalence of low (AMH <1 ng/mL) and very low (<0.5 ng/mL) ovarian reserve in BC survivors <40 years old, 12–24 months post-chemotherapy, to quantify the gonadotoxic impact of BC treatments.

**Methods:**

A systematic literature search of PubMed, Embase, and the Cochrane Library identified studies with AMH levels before and 12–24 months after chemotherapy in BC patients <40 years of age. Data on AMH levels were pooled using random-effects meta-analysis. Study quality was assessed using the Joanna Briggs Institute Critical Appraisal Checklist. This study is part of the FertiTOX project (www.fertitox.com).

**Results:**

Ten studies (860 BC survivors) were included. Mean AMH decline was −1.61 (95 % CI: -2.31; −0.91) post-chemotherapy. The pooled prevalence of AMH <1 ng/mL and <0.5 ng/mL was 58 % (46–70 %) and 53 % (41–64 %), respectively. High heterogeneity was observed (I^2^ >80 %).

**Conclusions:**

More than half of BC survivors have severely reduced ovarian reserve after chemotherapy, which is associated with a shortened fertile window and an increased risk of premature ovarian insufficiency. These findings highlight the need for pre-treatment fertility counseling and post-treatment ovarian insufficiency surveillance in routine oncology care.

## Highlights

• AMH levels drop from 3.79 ng/mL at BC diagnosis to 0.77 ng/mL after chemotherapy.
• > 50 % of patients have a very low ovarian reserve (AMH <0.5 ng/ml) post-treatment.
• BC chemotherapy imposes a high risk of premature ovarian insufficiency leading to infertility.
• Pre- and post-treatment fertility counseling is crucial for young patients.
• Follow-up for premature ovarian insufficiency should be part of routine oncology care.

## 1 Introduction

Worldwide, breast cancer (BC) is the most commonly diagnosed cancer disease and the leading cause of cancer-related death in women [1]. Thanks to advances in diagnosis and treatment, survival rates for women with BC during their reproductive years have steadily improved over the years. As a result of these positive developments, more and more women are not only surviving BC, but are returning to healthy and fulfilling lives. At the same time, the desire to have children is increasingly postponed in today's society [2]. The combination of improved cancer survival and later family planning has made the risk of infertility a key issue for the 5–7 %[3,4] of women affected by BC who are <40 years of age. A web-based survey of 657 young women with early breast cancer found that 56 % expressed a strong desire to have children in the future, while 73 % were concerned about potential infertility. In addition, 29 % of respondents reported that fertility concerns influenced their treatment decisions [5].

As a logical consequence, several guidelines recommend that patients should be counseled about the possibility of fertility preservation before starting BC therapies [[6], [7], [8]]. However, reliable prognostic parameters and data on the risk of infertility after chemotherapy to indicate the need for fertility preservation measures are limited. Amenorrhoea has long been used as a clinical indicator of reduced ovarian function after chemotherapy, but it only indicates a complete loss of ovarian reserve, is often difficult to interpret in breast cancer patients due to subsequent endocrine therapy, and is therefore not very reliable. The antral follicle count (AFC), on the other hand, also indicates the beginning of a decline in ovarian reserve, but requires a professional ultrasound scan and is therefore highly operator-dependent and therefore not sufficiently objective. Analysis of FSH levels is much more convenient, requiring only a simple blood test. However, FSH levels only rise when ovarian reserve is already very low. Furthermore, its cycle-dependent fluctuations make its interpretation difficult. Anti-Mullerian hormone (AMH) has been shown to be the best marker of ovarian reserve as it is not only sensitive, reliable and relatively stable over the menstrual cycle but also very convenient as it is measured in blood [[9], [10], [11], [12]].

As AMH concentrations vary considerably between individuals, it is difficult to define normal values [13]. However, low and very low levels have been defined to assess ovarian response to ovarian stimulation therapies[6,14] and to estimate the risk of premature ovarian insufficiency (POI) and the associated significant reduction in reproductive time [15,16]. The American College of Obstetricians and Gynaecologists (ACOG) and the American Society for Reproductive Medicine (ASRM) consider an AMH level <1 ng/mL to be a marker of decreased ovarian reserve [14]. The European Society of Human Reproduction and Embryology (ESHRE) follows the Bologna criteria when defining low ovarian reserve based on an AMH <0.5 ng/mL [6].

However, a meta-analysis providing data on the risk of low (defined as AMH <1 ng/mL) and very low (defined as AMH <0.5 ng/mL) ovarian reserve as a result of BC therapy has never been published. Therefore, as part of the FertiTOX project (www.fertitox.com), we performed such a meta-analysis to assess the risk of severe and very severe ovarian damage to fill this data gap in BC therapies [[17], [18], [19], [20], [21], [22], [23]].

## 2 Materials and methods

This study was conducted according to the Preferred Reporting Items for Systematic Reviews and Meta-Analyses (PRISMA) guidelines [24,25] and registered in PROSPERO, an international database of prospectively registered systematic reviews (CRD42023384042).

### 2.1 Research question and rationale for the search strategy

The clinical research question was formulated using the PICO model [26] to assess the risk of severe ovarian injury from BC-specific therapies in women aged <40 years.

(P) Patients: women <40 years of age at diagnosis of BC in a curable situation in whom AMH was measured both immediately before and at least 12 to max. 24 months after completion of chemotherapy. Exclusion criteria: breast cancer recurrence, breast cancer as a secondary malignancy and palliative situation.

(I) Intervention: chemotherapy.

(C) Comparison intervention: not applicable.

(O) Clinical outcome of interest: probability of occurrence of low and very low ovarian reserve defined by AMH <1 ng/mL or <0.5 ng/mL at least 12 to max. 24 months after completion of chemotherapy.

To assess the effect of cancer treatment on AMH levels during long-term follow-up, studies with a follow-up period of <12 months after the end of chemotherapy were excluded because of the possibility of residual effects of chemotherapy and therefore incomplete recovery of the ovarian reserve. Studies with a follow-up of more than 24 months after the end of chemotherapy were also excluded, because further ovarian recovery would not be expected at this time, and the chemotherapy-related adverse effect on ovarian reserve in the observed population of fertile women would be confounded by the age-related and therefore physiological decline in the follicle pool with increasing time since chemotherapy.

The decision to include only studies that reported AMH levels both before and after chemotherapy was based on the need for robust comparability of results between the included studies. Including only studies with AMH levels both before and after chemotherapy ensures that differences in baseline levels are included in the interpretation and thus minimises potential bias that could arise from looking only at absolute post-therapy levels, especially when these are strongly influenced by baseline levels, as is the case with AMH.

### 2.2 Literature search

To identify potentially relevant publications on the topic, a search strategy was developed and searched in MEDLINE, Embase and the Cochrane Library (see Supplementary File 1). After refinement, a librarian for specialist literature search (TK) set up the search strategy for each information source based on database-specific index terms and free text. The free text search included synonyms, acronyms and similar terms. In all databases, the built-in publication year filter was used to limit the results to the period 2000 to the present. In addition, in Cochrane the results were filtered for trials and systematic reviews. No other database-implemented restrictions on study types, languages or other formal criteria were applied in any of the sources. Animal-only trials were excluded from the searches using a double-negative search strategy based on Ovid's “human only” filters. The search was first performed on March 21, 2023 and updated on January 24, 2024. The results were deduplicated using the automated deduplication tool Deduklick [27] and updated using the “Bramer method” [28]. The results were imported into the Covidence screening tool [29], which identified and removed further duplicates.

The detailed final search strategies are presented in the appendix. In addition to electronic database searches, reference lists and bibliographies of relevant publications were reviewed for relevant studies.

### 2.3 Study selection

The search strategy identified a total of 6865 articles, of which 5789 were unique after duplicates were removed. 5789 titles, abstracts and 143 full texts were screened independently by authors MW, RS and AV using Covidence [29]. Disagreements were resolved in consultation with the third author, SW. All English, German or French language prospective or retrospective clinical studies were included that reported AMH levels both before and 12–24 months after breast cancer-specific chemotherapy in women aged <40 years in a curative setting.

### 2.4 Data extraction

Pre-defined relevant data from the 10 included papers were extracted independently by two researchers, MW and SW. Key variables included: study characteristics (study type, study duration, number of participants of interest), patient characteristics (age, tumor type), chemotherapy details (chemotherapy regimen, details of agents and doses used), and AMH levels before and 12–24 months after chemotherapy. AMH levels reported in pmol/L were converted to ng/mL using a conversion factor of 7.14.

### 2.5 Data synthesis and statistical analyses

The objective of this systematic review and meta-analysis was to determine the risk of low and very low ovarian reserve, defined as AMH <1 ng/mL or <0.5 ng/mL 12–24 months after the end of chemotherapy, in breast cancer patients aged <40 years. The incidence of gonadal toxicity was calculated by dividing the number of patients who met the criteria for gonadal toxicity by the number of patients at potential risk for this outcome in each study. If the exact results according to our definition of low and very low ovarian reserve were explicitly reported in a publication, we used these exact values. However, when post-therapeutic AMH levels were reported as a mean or median, we interpreted the data in terms of the estimated frequency of an AMH level <1 ng/mL or <0.5 ng/mL. In such cases, the term “at least” was used to determine the minimum proportion of patients with an AMH level <1 ng/mL or <0.5 ng/mL based on the reported statistics (e.g., with a median AMH level of 0.06 ng/mL (range 0.01–4.76) 12 months after chemotherapy in n = 44, it was concluded that at least n = 22 and thus 50 % of patients had an AMH level <1 ng/mL [30]). It is likely that the true proportion of patients with an AMH level <1 ng/mL is higher, as both the mean and the median are conservative estimates. The metafor function in R software (R Core Team, Vienna, Austria, 2013) was used to analyse the pooled prevalence. Cohen's Q and I2 statistics were used to assess heterogeneity. Random effects models were selected in cases of high heterogeneity.

In a second analysis, the treatment effect, defined as the difference between AMH before and after chemotherapy, was calculated for each included study. The variance of the treatment effect, derived from the standard deviations or standard errors of the paired differences between AMH levels, was calculated. The mean effect size was calculated using the inverse variance method in random effect models. As the absolute AMH values, including their standard deviations, were not available for all publications in the first analysis, only six publications could be included in the second analysis.

### 2.6 Quality assessment

The quality of each study was assessed using the Joanna Briggs Institute Critical Appraisal Checklist for Cohort Studies [31]. Items were scored as “yes” (1 point), “no” (−1 point), “unclear” (−1 point), and “not applicable” (0 points). Studies with a total score of 9–11 points were considered to be of high quality, those with a score of 6–8 points were considered to be of moderate quality, and those with a score of 0–5 points were considered to be of low quality. All included studies were independently assessed for risk of bias by the authors MW and RS. Disagreements were resolved by consensus. The rating of each study is shown in Table 1.

**Table 1:**
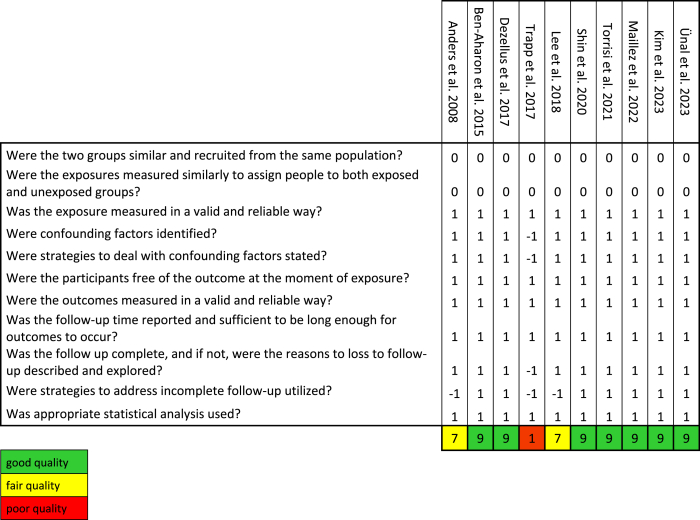
Quality assessment using the Joanna Briggs Institute Cohort Study Checklist. Individual items were scored as “yes” (1 point), “no” (−1 point), “unclear” (−1 point), and “not applicable” (0 points). Studies with a total score of 9–11 points were considered to be of high quality, those with a score of 6–8 points were considered to be of moderate quality, and those with a score of 0–5 points were considered to be of low quality.

## 3 Results

### 3.1 Results of the systematic review

**Fig. 1:**
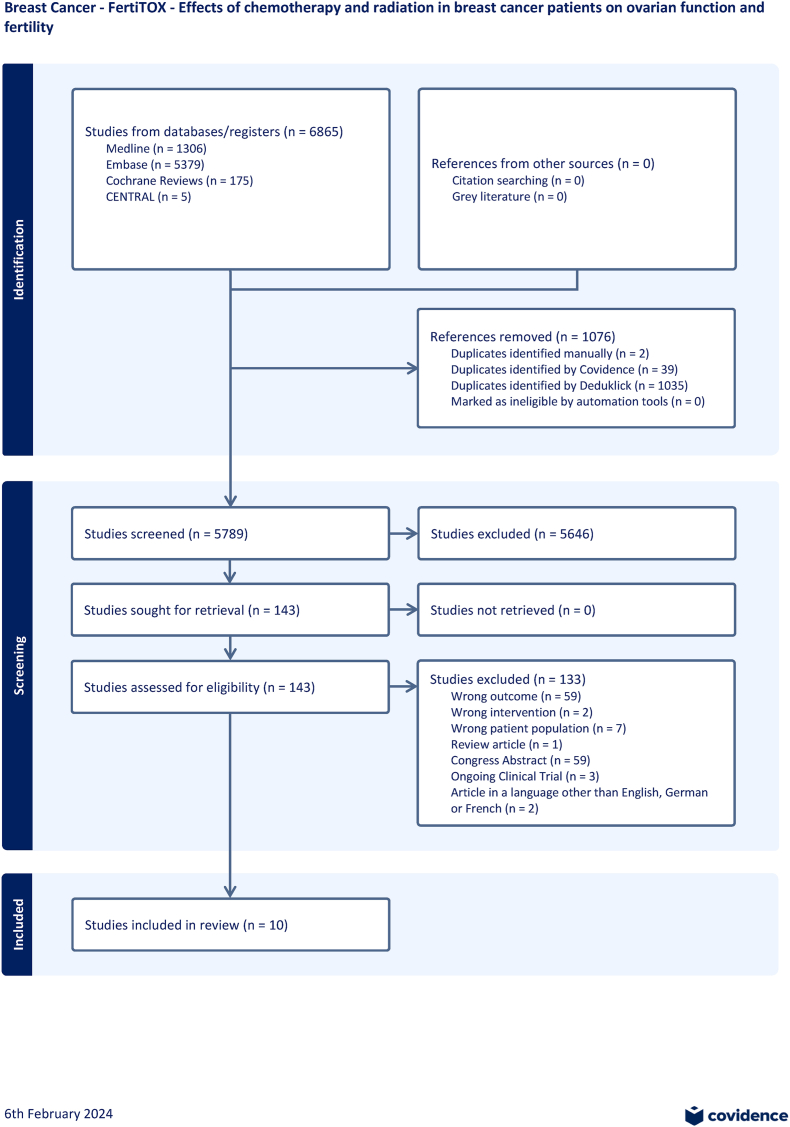
PRISMA flow diagram.

After reviewing titles, abstracts and full texts, 10 studies from 7 different countries (Korea: n = 3, France: n = 2, Germany: n = 1, Israel: n = 1, Italy: n = 1, Turkey: n = 1, USA: n = 1) met our inclusion criteria and were subjected to data extraction. (see PRISMA flow diagram, Fig. 1).

### 3.2 Study characteristics

The main study characteristics and results of the 10 included studies are shown in Table 2. The analysis included 9 prospective cohort studies [30,[32], [33], [34], [35], [36], [37], [38], [39]] and 1 randomised controlled trial [40] conducted between 2005 and 2019 and published between 2008 and 2023. A total of 860 subjects met the inclusion criteria and were included in the analysis. The sample size of each study ranged from 3 [32] to 193 [35] subjects with a follow-up period of 1–5 years.

**Table 2:** Main study characteristics and outcomes of the included studies Symbols and abbreviations: x = no data.

First author, Journal, Year of publication	Study design & duration	Identification of participants of interest	Age of participants of interest at time of diagnosis/therapy (years)	AMH pre-chemotherapy	AMH post-chemotx (12–24 months after chemotherapy)	AMH <1 ng/mL 12–24 months after chemotherapy	AMH <0.5 ng/mL 12–24 months after chemotherapy
Anders C. et al., Cancer Invest., 2008	Prospective cohort study x	n = 10 <35 yrs pre-chemotherapy **n=3** post-chemotx	n = 10 range 21–34	n = 10 median 2.72 ng/mL	n = 3 median 2.72 ng/mL	at least 2/3 (12 months post-chemotherapy) **at least 67 %**	at least 2/3 (12 months post-chemotherapy) **at least 67 %**
Ben-Aharon I. et al., Oncologist, 2015	Prospective cohort study July 2009 - March 2011	**n=20**	n = 20 median 34 ± 5.24, range 26–43 (2/20 >40: 42 & 43)	n = 20 mean 1,2 ng/mL	n = 20 mean 0.4 ng/mL	at least 10/20 (12 months post-chemotherapy) **at least 50 %**	at least 10/20 (12 months post-chemotherapy) **at least 50 %**
Dezellus A. et al., Eur J Cancer, 2017	Prospective cohort study January 2010 - July 2011	n = 249 pre-chemotherapy **n=181** post-chemotherapy	n = 249 mean 34.8 ± 3.9, range 18–39	n = 249 mean 4.19 ± 4.84 ng/mL, median 2.95	n = 181 mean 0.78 ± 1.4 ng/mL	at least 96/181 (24 months post-chemotherapy) **at least 53 %**	at least 96/181 (24 months post-chemotherapy) **at least 53 %**
Trapp E. et al., Breast, 2017	RCT September 2005 - March 2007	n = 170 pre-chemotherapy **n=101** post-chemotherapy	n = 170 median 36, range 21–40, mean 35.8	n = 170 median 1.37 ± 2.12 ng/mL, range <0.1–11.3	n = 101 mean <0.1 ± 0.46 ng/mL, range <0.1–3.9	97/101 (24 months post-chemotherapy) **96 %**	at least 74/101 (24 months post-chemotherapy) **at least 73 %**
Lee D. et al., Breast Cancer Res Treat, 2018	Prospective cohort study January 2013 - December 2014	**n=105**	n = 105 mean 32.3 ± 3.9, range 23–42	n = 75 with AMH ≥1 ng/mL 12 months post-chemotherapy had 5.9 ± 2.9 ng/mL pre-chemotherapy n = 30 with AMH <1 ng/mL 12 months post-chemotherapy had 2.3 ± 2.5 ng/mL pre-chemotherapy	n = 75 with AMH ≥1 ng/mL n = 30 with AMH <1 ng/mL	30/105 (12 months post chemotherapy) **29 %**	x
Shin J. et al., J Breast Cancer, 2020	Prospective cohort study October 2009 - February 2016	n = 136 pre-chemotherapy **n=95** post-chemotherapy	n = 136 median 32, range 19–39	n = 136 mean 5.6 ± 0.4 ng/mL	n = 95 mean 1.47 ± 1.93 ng/mL	53/95 (12 months post-chemotherapy) **55.8 %**	x
Torrisi R. et al., Breast Care (Basel), 2022	Prospective cohort study July 2013 - June 2015	n = 40 pre-chemotherapy **n=31** post-chemotherapy	n = 40 median 36, range 28–40	n = 31 mean 2.36 ± 1.8 ng/mL median 2.38, range 0.39–8.13	n = 31 mean 0.02 ± 0.02 ng/mL median 0.19, range 0.01–1.12	at least 29/31 (12 months post-chemotherapy) **at least 93.5 %**	at least 16/31 (12 months post-chemotherapy) **at least 51.6 %**
Maillez A. et al., Int J Cancer, 2022	Prospective cohort study September 2011 - December 2016	n = 126 pre-chemotherapy **n=87** post-chemotherapy	n = 126 mean 31.2 ± 3.4 median 32, range 23–37	n = 126 mean 4.34 ± 4.38 ng/mL	n = 87 mean 0.73 ± 0.77 ng/mL median 0.5, range 0.07–3.81	at least 29/87 (12–24 months post-chemotherapy) **at least 33 %**	at least 29/87 (12–24 months post-chemotherapy) **at least 33 %**
Kim S. et al., Breast Cancer Res Treat, 2023	Prospective cohort study 2016–2019	**n=193**	n = 193 mean 33.6 (all ≤40)	n = 193 mean 4.2 ± 2.1 ng/mL	n = 193 mean 1.2 ± 1.4 ng/mL	102/193 (12 months post-chemotherapy) **53 %**	x
Ünal C. et al., Curr Oncol, 2023	Prospective cohort study x	**n=44**	n = 44 range 23 - ≤ 40	n = 44 mean 1.97 ± 1.61 ng/mL median 1.52, range 0.03–6.20	n = 44 mean 0.54 ± 1.04 ng/mL median 0.06, range 0.03–4.33	at least 22/44 (12 months post-chemotherapy) **at least 50 %**	at least 22/44 (12 months post-chemotherapy) **at least 50 %**

### 3.3 Quality assessment

Most studies were rated as having good methodological quality (n = 9), while two studies were rated as fair (n = 2) and one study as poor (n = 1) (see Table 1). The main reason for the fair quality rating was the lack of strategies to deal with incomplete follow-up. The study rated as poor quality failed to meet several methodological criteria.

### 3.4 Patient characteristics

9/10 studies [[30], [32], [33], [34], [35], [36], [37], [39], [40]] included subjects with newly diagnosed breast cancer FIGO stages I-III. 1/10 studies [38] included women with breast cancer FIGO I-IV, with only 2.2 % of subjects having FIGO IV tumor stage. Subjects were aged between 18 and 40 years (mean 34.2 years) (see Table 2).

### 3.5 Therapy characteristics

#### 3.5.1 Chemotherapy

Polychemotherapy regimens - as typically used in BC - included alkylating agents (98 %), antracyclines (97 %), taxanes (78 %), antimetabolites (42 %), and platinum derivatives (1 %) (see Table 3). Subgroup analyses of the gonadotoxic effects of individual chemotherapeutic agents or specific chemotherapy regimens and doses were not possible due to insufficient data. Similarly, the influence of targeted therapies and endocrine therapies could not be analysed separately. Furthermore, the effect of fertility preservation strategies such as ovarian function suppression (OFS) with gonadotropin-releasing hormone agonists (GnRHa) or ovarian tissue cryopreservation could not be assessed due to a lack of detailed data in the included studies (see Table 4).

**Table 3:** Chemotherapeutic agents in the included studies. Symbols and abbreviations: PaOfIn = patients of interest.

First author, Journal, year	Antracyclines	Alkylating agents	Taxanes	Antimetabolites	Platinum derivatives
Anders C., Cancer Invest., 2008	100 % PaOfIn n = 3	100 % PaOfIn n = 3	68 % PaOfIn n = 2	11 % PaOfIn n = 0	2 % PaOfIn n = 0
Ben-Aharon I., Oncologist, 2015	85 % PaOfIn n = 17	65 % PaOfIn n = 13	85 % PaOfIn n = 17	25 % PaOfIn n = 5	35 % PaOfIn n = 7
Dezellus A., Eur J Cancer, 2017	94.4 % PaOfIn n = 171	94 % PaOfIn n = 171	95.2 % PaOfIn n = 172	93.6 % PaOfIn n = 169	0 %
Trapp E., Breast, 2017	100 % PaOfIn n = 101	100 % PaOfIn n = 101	100 % PaOfIn n = 101	100 % PaOfIn n = 101	0 %
Lee D., Breast Cancer Res Treat, 2018	100 % PaOfIn n = 105	100 % PaOfIn n = 105	59.1 % PaOfIn n = 62	23.8 % PaOfIn n = 25	0 %
Shin J., J Breast Cancer, 2020	97.8 % PaOfIn n = 93	100 % PaOfIn n = 95	48.5 % PaOfIn n = 46	50.7 % PaOfIn n = 48	0 %
Torrisi R., Breast Care (Basel), 2022	100 % PaOfIn n = 31	100 % PaOfIn n = 31	29.0 % PaOfIn n = 9	29.0 % PaOfIn n = 9	0 %
Maillez A., Int J Cancer, 2022	100 % PaOfIn n = 87	100 % PaOfIn n = 87	100 % PaOfIn n = 87	0 %	0 %
Kim S., Breast Cancer Res Treat, 2023	100 % PaOfIn n = 193	100 % PaOfIn n = 193	77.2 % PaOfIn n = 149	0 %	0 %
Ünal C., Curr Oncol, 2023	76.1 % PaOfIn n = 33	94.4 % PaOfIn n = 42	62 % PaOfIn n = 27	0 %	0 %
	PaOfIn n = 834 97 %	PaOfIn n = 841 98 %	PaOfIn n = 672 78 %	PaOfIn n = 357 42 %	PaOfIn n = 7 1 %

**Table 4:** Type of adjuvant therapies and fertility preservation techniques used in the included trials. Symbols and abbreviations: PaOfIn = patients of interest, x = no data.

First author, Journal, year	Trastuzumab adjuvant	Tamoxifen adjuvant	Aromatase inhibitor adjuvant	GnRH adjuvant	GnRH for Ovarian Function Suppression	Oocyte/embryo cryopreservation pre-chemothx	Ovarian tissue cryopreservation pre-chemothx
Anders C., Cancer Invest., 2008	18 % PaOfIn n = 1	59 % PaOfIn n = 2	2 % PaOfIn n = 0	x	0 %	x	x
Ben-Aharon I., Oncologist, 2015	35 % PaOfIn n = 7	x	x	x	50 % PaOfIn n = 10	65 % PaOfIn n = 13	x
Dezellus A., Eur J Cancer, 2017	27.3 % PaOfIn n = 49	60.2 % PaOfIn n = 109	x	x	3.6 % PaOfIn n = 7	x	2 % PaOfIn n = 4
Trapp E., Breast, 2017	x	62 % PaOfIn n = 63	53 % PaOfIn n = 54	53 % PaOfIn n = 54	7.1 % PaOfIn n = 7	x	x
Lee D., Breast Cancer Res Treat, 2018	x	67.6 % PaOfIn n = 71	x	x	100 % PaOfIn n = 105	9.5 % PaOfIn n = 10	0 %
Shin J., J Breast Cancer, 2020	15.2 % PaOfIn n = 14	75 % PaOfIn n = 71	0 %	x	100 % PaOfIn n = 95	x	x
Torrisi R., Breast Care (Basel), 2022	x	74.2 % PaOfIn n = 23	80 % PaOfIn n = 25	x	100 % PaOfIn n = 31	x	x
Maillez A., Int J Cancer, 2022	22,2 % PaOfIn n = 19	48.4 % PaOfIn n = 42	0 %	0 %	0 %	46 % PaOfIn n = 40	x
Kim S., Breast Cancer Res Treat, 2023	x	x	0 %	0 %	100 % PaOfIn n = 193	x	x
Ünal C., Curr Oncol, 2023	x	x	x	x	x	x	x
	At least 10.5 % PaOfIn n = 90	At least 44.3 % PaOfIn n = 381	At least 9.2 % PaOfIn n = 79	At least 6.3 % PaOfIn n = 54	At least 52.1 % PaOfIn n = 448	At least 7.3 % PaOfIn n = 63	At least 0.5 % PaOfIn n = 4

### 3.6 Risk of low and very low ovarian reserve

**Fig. 2:**
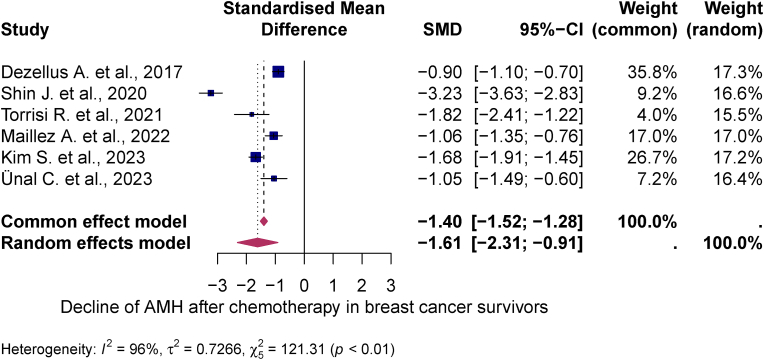
Meta-analysis of therapy-related AMH decline in breast cancer survivors This meta-analysis illustrates the therapy-related decline in anti-Müllerian hormone (AMH) after chemotherapy in breast cancer survivors. The figure shows the standardized mean differences (SMD) from different studies with their 95 % confidence intervals. Both a common-effects and a random-effects model are included, with high heterogeneity observed (I^2^ = 96 %), indicating substantial variation between studies.

**Fig. 3:**
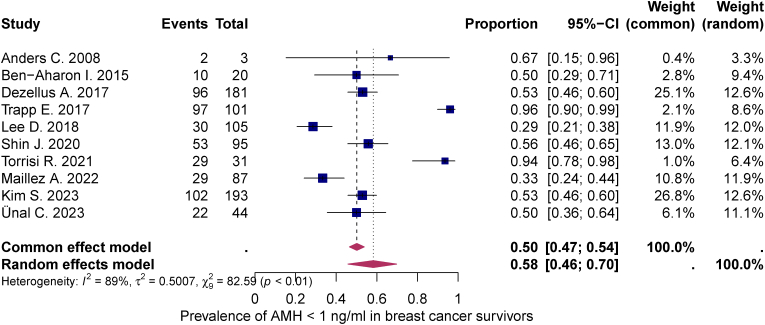
Meta-analysis of post-treatment prevalence of AMH <1 ng/mL in breast cancer survivors This meta-analysis shows the prevalence of post-treatment AMH levels below 1 ng/ml in breast cancer survivors. The figure shows proportions from multiple studies with their respective 95 % confidence intervals. Both a common-effects and a random-effects model are included, with high heterogeneity observed (I^2^ = 89 %), indicating considerable variation between studies.

**Fig. 4:**
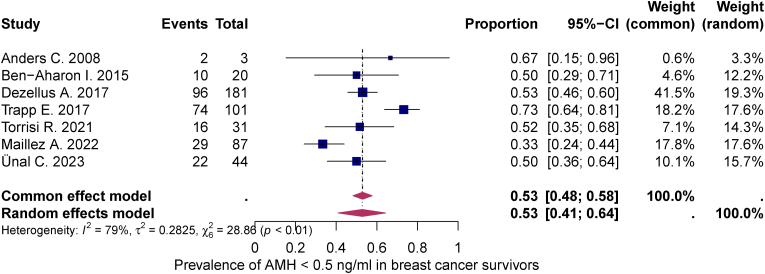
Meta-analysis of post-treatment prevalence of AMH <0.5 ng/mL in breast cancer survivors This meta-analysis examines the prevalence of post-treatment (AMH) levels below 0.5 ng/ml in breast cancer survivors. The figure shows proportions from multiple studies with their 95 % confidence intervals. Both a common effect and a random effect model are included, with substantial heterogeneity observed (I^2^ = 79 %), indicating moderate variation between studies.

Quantitative synthesis was performed in all 10 studies. At BC diagnosis, the mean AMH level was 3.79 ng/mL (95 % CI: 2.69; 4.89). 12–24 months after chemotherapy, it had fallen to 0.77 ng/mL (95 % CI: 0.37; 1.18). The statistically significant decrease of −1.61 (95 % CI: -2.31; −0.91) in the mean AMH concentration 12–24 months after completion of chemotherapy lead to a pooled prevalence of low (AMH <1 ng/mL) and very low (AMH <0.5 ng/mL) ovarian reserve (95 % CI) in at least 58 % (46–70 %) and 53 % (41–64 %) of the study population, respectively. The heterogeneity test showed significant heterogeneity between studies I^2^ = 89 %, p < 0.01 and I^2^ = 81 %, p < 0.01 (see Fig. 2, Fig. 3, Fig. 4).

## 4 Discussion

This systematic review and meta-analysis revealed a significant impact of BC-specific chemotherapy on ovarian reserve in breast cancer survivors of childbearing age. Chemotherapy substantially reduced the AMH levels, resulting in very low ovarian reserve in more than half of the patients studied 12–24 months after completion of chemotherapy.

The strengths of this review and meta-analysis lie in its systematic methodology, strict study selection, and the use of AMH as the primary biomarker to quantify gonadotoxic effects. By including studies that reported AMH levels both before and after chemotherapy, potential bias was minimized.

The limitation is the high heterogeneity (I^2^ > 80 %) of the included studies, which indicates significant differences between studies and limits the generalizability of the results. This could be due to the inclusion of studies with significant differences in patient characteristics (e.g., age, pre-treatment AMH levels, BRCA mutation carriers), differences in polychemotherapy regimens and dosages, differences in the frequency and type of endocrine and targeted therapies, and methodological differences in AMH determination. Another limitation is that subgroup analyses regarding the gonadotoxic effects of individual chemotherapeutic agents or specific chemotherapy regimens and doses could not be performed. Although alkylating agents, anthracyclines, taxanes, and antimetabolites were commonly used in the included studies, the variation in the total of 25 treatment combinations and dosages prevented us from isolating the specific effects of each single drug or chemotherapy regimen. A subgroup analysis of the potential modifying effects of endocrine therapies on AMH levels, such as selective estrogen receptor modulators (SERMs, e.g., tamoxifen) and the combination of GnRHa and aromatase inhibitors (AIs, e.g., letrozole) or tamoxifen was also not be possible.

As amenorrhoea as a clinical indicator of reduced ovarian function after chemotherapy is not very reliable, AMH concentrations are increasingly used to estimate the impact of gonadotoxic therapies on ovarian reserve. However, the specific effects of individual chemotherapy agents and regimens on actual ovarian reserve as measured by AMH and long-term fertility are less well understood.

The decrease in AMH concentration as a result of BC therapy has already been analysed previously. Our results of a mean reduction in AMH concentration of −1.61 (95 % CI: -2.31, −0.91) are in consistency with previous studies. Romito et al. used AMH as a parameter to estimate the absolute reduction in ovarian reserve. They found that women aged 30–35 and 35–40 years showed a significant decrease in AMH levels one year after chemotherapy, with a mean reduction of −2.73 (95 % CI: -3.77, −1.70) and −2.69 (95 % CI: -2.87, −2.50), respectively. While the meta-analysis included only two studies in patients aged 35–40 years, the analysis in patients aged 30–35 years was based on four studies [41]. Despite these consistent findings of chemotherapy-induced reduction in ovarian reserve, both in the meta-analysis by Romito et al. and in our own study, the available data are too limited to reliably quantify valid subgroup analyses on the gonadotoxicity of specific chemotherapeutic agents, individual treatment regimens, let alone the influence of targeted or endocrine therapies on ovarian function.

Among the studies included in our meta-analysis, the highest prevalence of posttherapeutic AMH levels <1 ng/mL was reported in the study by Trapp et al. (2017) [40] with 96 % (95 % CI: 90–99 %) and Torrisi et al. (2021) [39] with 94 % (95 % CI: 78–98 %). In contrast, the lowest prevalence was reported in the study by Lee et al. (2018) [36] with 29 % (95 % CI: 21–38 %) and Maillez et al. (2022) [37] with 33 % (95 % CI: 34–44 %). Regarding the meta-analysis of the prevalence of post-therapeutic AMH levels <0.5 ng/mL, the extremes on both sides are the study by Trapp et al. (2017) [40] with 73 % (95 % CI: 64–81 %) and the study by Maillez et al. (2022) [37] with 33 % (95 % CI: 34–44 %). As shown in Table 3, the four studies [36,37,39,40] with the most extreme results show no discernible differences in the drug combinations used that could explain the different gonadotoxicity in both directions.

These findings raise the question of whether and to what extent low AMH concentrations may be due to endocrine treatments. Existing data suggest that adjuvant tamoxifen, taken at the typical dose of 20 mg/day, has no ovarian stimulating effect and therefore does not affect AMH interpretation. [34,42,43] However, GnRHa leads to a decrease in AMH concentration, which is dependent on factors such as polycystic ovary syndrome, obesity, age, and duration of medication [44]. Because of these different factors influencing AMH levels, and because the effect of long-term adjuvant endocrine therapy with GnRHa in combination with AIs or SERMs is poorly understood, the quantitative effect of GnRHa on AMH concentration cannot be estimated, making interpretation difficult. Although this review was unable to analyse the influence of adjuvant therapies, such as endocrine treatment or fertility preservation techniques, on AMH levels quantitatively, Table 4 provides an overview of their use in the included studies. The frequent concomitant administration of AI, tamoxifen and/or GnRH agonists (GnRHa) emphasises the need to consider their potential impact on AMH levels when evaluating ovarian reserve post-treatment. Furthermore, the limited documentation of fertility preservation strategies emphasises the ongoing discrepancy between guideline recommendations and clinical practice.

Another methodological issue of our systematic review and meta-analysis is the interpretation of AMH levels as a surrogate marker of fertility. Although AMH correlates with the quantity but not the quality of ovarian reserve, it is not a direct predictor of spontaneous pregnancy rates and live births. Consequently, in an eumenorrhoeic woman with no history of subfertility, AMH provides no information about the likelihood of conception or the time to conception, regardless of the measurement result. Thus, eumenorrhoeic women with low AMH levels do not differ in fertility from women with high AMH levels [[45], [46], [47]]. The best criterion for estimating the likelihood of spontaneous pregnancy is still age, as it correlates strongly with the risk of genetic abnormalities in the oocyte. This in turn is a major factor in the failure to conceive or the increased risk of early miscarriage [48]. However, measuring AMH levels still has relevant prognostic value for fertility as it may indicate a shortened reproductive window [15,16]. A nested case-control study within the prospective Nurses' Health Study II cohort found a significant association between lower AMH levels in women aged 32–44 years and an increased risk of early natural menopause. This study defined an AMH threshold of 1 ng/mL for women aged 35 years to predict the risk of menopause before the age of 45 years, achieving a sensitivity of 64 % and a specificity of 73 % [49].

In this context, AMH measurement should be considered approximately one year after completion of gonadotoxic therapy, even if the menstrual cycle is normal, in order to advise the young woman individually about her ovarian reserve, the remaining reproductive window and the risk of POI or early menopause. In a prospective cohort study of perimenopausal women (mean age 42 years ± SD 2.7), Sowers et al. showed that AMH drops to undetectable levels approximately 5 years before menopause, corresponding to the last menstrual period [50]. However, caution should be exercised when extrapolating these results to a younger patient population, as the age-dependent dynamics of AMH decline are very different. Rather, Freeman et al. showed that in younger women aged 35–39 years, an undetectable AMH level is associated with a median time to menopause of almost 10 years - a period significantly longer than in the fifth decade of life with comparable AMH levels [51]. To reliably estimate the remaining potentially fertile window, the AMH level must always be interpreted in the context of the patient's age, i.e. AMH has limited value in accurately predicting the individual age of menopause. However, a low AMH level <1 ng/mL in young patients indicates an increased risk of POI or early menopause before the age of 45.

## 5 Conclusion

BC chemotherapy has a significant relevant impact on ovarian reserve in survivors of childbearing age. These findings are of high clinical relevance, as they not only result in a shortened reproductive lifespan, but also significantly increase the risk of premature ovarian insufficiency (POI) and early menopause, which in turn have a negative impact on long-term health. This highlights the need to integrate pre- and post-treatment counseling on fertility and fertility preservation strategies as well as post-treatment follow-up on ovarian insufficiency into routine oncology care.

## CRediT authorship contribution statement

**Susanna Weidlinger:** Writing – review & editing, Writing – original draft, Data curation. **Magdalena Weidlinger:** Data curation. **Rose-Maria Schramm:** Data curation. **Angela Vidal:** Validation. **Janna Pape:** Data curation. **Tanya Karrer:** Resources. **Manuela Rabaglio:** Resources. **Michael von Wolff:** Methodology, Conceptualization.

## Declaration of competing interest

The authors have stated that there are no conflicts of interest in connection with this article.

## Study funding

The study was supported by a grant from the Swiss Cancer League grant (Grant number: KLS-5650-08-2022).
