# Supplementary material for: High impact of chemotherapy on ovarian reserve in breast cancer survivors of reproductive age: A systematic review and meta-analysis
Source: Breast. 2025 Jun 13;82:104514. doi: 10.1016/j.breast.2025.104514 (PMC12221882; doi:10.1016/j.breast.2025.104514)
Supplement: Multimedia component 1 [file mmc1.docx]

**Search Strategies**

**Detailed search strategy**

To identify potentially relevant publications on the topic, a search strategy was designed, and investigated in MEDLINE, Embase, and Cochrane Library. A medical information specialist developed an initial search strategy in Embase and tested it against a list of core references to ensure key publications were included. After refinement, the information specialist set up the search strategy for each information source based on database-specific index terms and free text. The free text search included synonyms, acronyms, and similar terms. In all databases the in-built publication year filter was applied to limit the results for the period from 2000 to present. Additionally, in Cochrane the results were filtered by trials and systematic reviews. No other database-provided limits have been applied in any sources considering study types, languages or any other formal criteria. Studies concerning exclusively animals were excluded from the searches by using a double-negative search strategy based on the "Humans only" filters by Ovid. The search was first run on 21/03/2023 and updated on 24/01/2024. The results were deduplicated using the automated deduplication tool Deduklick (https://www.risklick.ch/products/deduklick/)^[[1]](#footnote-1)^. The update was carried out by following the “Bramer method”^[[2]](#footnote-2)^. We imported the results into the Covidence screening tool (https://www.covidence.org/). Covidence has detected and removed a further 39 duplicates.

| **Search date** | **Database searched** | **Platform** | **Years of coverage** | **Records** |
| --- | --- | --- | --- | --- |
| 24 January 2024 | Embase | Ovid | 1974 - 2024 January 23 | 5379 |
| 24 January 2024 | Medline | Ovid | 1946 - 2024 January 23 | 1306 |
| 24 January 2024 | Cochrane Database of Systematic Reviews | Wiley | 1992 - present | 175 |
| 24 January 2024 | Cochrane Central Register of Controlled Trials | Wiley | 1992 - present | 5 |
|  |  |  | Sum of references | 6865 |
|  |  |  | Removed duplicates | - 1076 |
|  |  |  | **Total references** | **5789** |

**Database Search Strategies**

**Search date: January 24, 2024**

********************************************

**Ovid MEDLINE(R) ALL <1946 to January 23, 2024>**

Search date: 24/01/2024

1 (((Breast* or mammary or lobular) adj3 (cancer* or neoplas* or tumo?r* or carcin* or malign* or adenocarcin* or adeno-carcin*)) or IBC or DCIS or LCIS or breastcancer?).ti,ab,kf. or exp breast tumor/ 501941

2 (chemotherap* or chemo-therap* or chemoradiotherap* or chemo-radiotherap* or adjuvant drug therap* or carcinochemotherap* carcino-chemotherap* or antineoplastic agent* or anti-neoplastic agent* or antineoplastic drug* or anti-neoplastic drug* or antitumo?r agent* or anti-tumo?r agent* or antitumo?r drug* or anti-tumo?r drug* or anticancer* agent* or anti-cancer* agent* or anticancer* drug* or anti-cancer drug* or anticarcinogen* or anti-carcinogen* or anticancerogen* or anti-cancerogen* or ((cancer* or tumo?r* or neoplas*) adj3 treat*)).ti,ab,kf. 892993

3 exp Antineoplastic Agents/ or exp Combined Modality Therapy/ or exp Antineoplastic Combined Chemotherapy Protocols/ or exp Chemotherapy, adjuvant/ or exp Antineoplastic Protocols/ or exp chemoradiotherapy/ or Radioimmunotherapy/ 1508335

4 (Radio-therap* or radiotherap* or radiationtherap* or chemoradiotherap* or radiochemotherap* or protontherap* or radiosurg* or radio-surg* or irradiation* or x-ray-therap* or therap* radiolog* or IMRT* or IORT* or radioimmunotherap* or radio-immuno-therap* or ((radiat* or irradiat* or radioisotope* or radio-isotope* or chemoradio or chemo-radio or radiochemo or radio-chemo or proton or x-ray or xray) adj2 (therap* or oncolog* or brachytherap* or brachy-therap*))).ti,ab,kf. 532798

5 exp Radiotherapy/ or exp Radiotherapy, Adjuvant/ 209793

6 (((biologic* response modifier* or BRM? or immune or imune or immunogenic or immunoglobulin or immune-globulin or immunological or immunomodul* or immune-modul* or immunomodur*) adj2 (therap* or treatment or intervention or drug* or agent*1)) or immunotherap* or imunotherap* or "immunity modulator*").ti,ab,kf. 190059

7 Immunotherapy/ 67069

8 (AMH or Anti-Mu?llerian Hormone* or Antimu?llerian Hormone* or Anti-Mu?llerian Factor* or Mu?llerian Inhibiting Hormone* or mu?llerian inhibitor* or testis or testes or testicle* or spermatogenes* or sperm* or semen or gametogenes*).ti,ab,kf. 245444

9 exp Fertility/ or exp Infertility/ or exp Gonads/ or menopause, premature/ or Reproduction/ or Gametogenesis/ or Spermatozoa/ 372653

10 or/2-7 2474217

11 or/8-9 490535

12 1 and 10 and 11 1924

13 (exp animals/ or exp animal experimentation/ or exp models, animal/ or exp plants/ or exp fungi/) not humans/ 5626837

14 12 not 13 1758

15 limit 14 to yr="2000-current" 1306

********************************************

**Embase <1974 to 2024 January 23>**

Search date: 24/01/2024

1 (((Breast* or mammary or lobular) adj3 (cancer* or neoplas* or tumo?r* or carcin* or malign* or adenocarcin* or adeno-carcin*)) or IBC or DCIS or LCIS or breastcancer?).ti,ab,kf. 622214

2 exp breast tumor/ 664617

3 or/1-2 767166

4 (chemotherap* or chemo-therap* or chemoradiotherap* or chemo-radiotherap* or adjuvant drug therap* or carcinochemotherap* carcino-chemotherap* or antineoplastic agent* or anti-neoplastic agent* or antineoplastic drug* or anti-neoplastic drug* or antitumo?r agent* or anti-tumo?r agent* or antitumo?r drug* or anti-tumo?r drug* or anticancer* agent* or anti-cancer* agent* or anticancer* drug* or anti-cancer drug* or anticarcinogen* or anti-carcinogen* or anticancerogen* or anti-cancerogen* or ((cancer* or tumo?r* or neoplas*) adj3 treat*)).ti,ab,kf. 1346079

5 exp antineoplastic agent/ or exp multimodality cancer therapy/ or exp cancer chemotherapy/ or exp antineoplastic protocol/ 3190745

6 (Radio-therap* or radiotherap* or radiationtherap* or chemoradiotherap* or radiochemotherap* or protontherap* or radiosurg* or radio-surg* or irradiation* or x-ray-therap* or therap* radiolog* or IMRT* or IORT* or radioimmunotherap* or radio-immuno-therap* or ((radiat* or irradiat* or radioisotope* or radio-isotope* or chemoradio or chemo-radio or radiochemo or radio-chemo or proton or x-ray or xray) adj2 (therap* or oncolog* or brachytherap* or brachy-therap*))).ti,ab,kf. 733210

7 exp cancer radiotherapy/ 340868

8 (((biologic* response modifier* or BRM? or immune or imune or immunogenic or immunoglobulin or immune-globulin or immunological or immunomodul* or immune-modul* or immunomodur*) adj2 (therap* or treatment or intervention or drug* or agent*1)) or immunotherap* or imunotherap* or "immunity modulator*").ti,ab,kf. 294557

9 exp immunotherapy/ 326086

10 or/4-9 4318128

11 (AMH or Anti-Mu?llerian Hormone* or Antimu?llerian Hormone* or Anti-Mu?llerian Factor* or Mu?llerian Inhibiting Hormone* or mu?llerian inhibitor* or testis or testes or testicle* or spermatogenes* or sperm* or semen or gametogenes*).ti,ab,kf. 287467

12 exp fertility/ or exp infertility/ or exp semen analysis/ or exp gonad/ or exp early menopause/ or reproduction/ or gemetogenesis/ or spermatozoon/ 569200

13 or/11-12 680891

14 3 and 10 and 13 6184

15 (exp animal/ or exp invertebrate/ or nonhuman/ or animal experiment/ or animal tissue/ or animal model/ or exp plant/ or exp fungus/) not (exp human/ or human tissue/) 7833192

16 14 not 15 5793

17 limit 16 to yr="2000-current" 5379

********************************************

**Cochrane Library <1996 to present>**

Search date: 24/01/2024

#1 (((Breast* or mammary or lobular) NEAR/3 (cancer* or neoplas* or tumor* tumour* or carcin* or malign* or adenocarcin* or adeno-carcin*) or IBC or DCIS or LCIS or breastcancer*)):ti,ab,kw 45951

#2 [mh "Breast Neoplasms"] 18236

#3 #1 or #2 45951

#4 (chemotherap* or chemo-therap* or chemoradiotherap* or chemo-radiotherap* or (adjuvant NEXT drug NEXT therap*) or carcinochemotherap* carcino-chemotherap* or (antineoplastic NEXT agent*) or (anti-neoplastic NEXT agent*) or (antineoplastic NEXT drug*) or (anti-neoplastic NEXT drug*) or (antitumor NEXT agent*) or (antitumour NEXT agent*) or (anti-tumor NEXT agent*) or (anti-tumour NEXT agent*) or (antitumor NEXT drug*) or (antitumour NEXT drug*) or (anti-tumor NEXT drug*) or (anti-tumour NEXT drug*) or (anticancer* NEXT agent*) or (anti-cancer* NEXT agent*) or (anticancer* NEXT drug*) or (anti-cancer NEXT drug*) or anticarcinogen* or anti-carcinogen* or anticancerogen* or anti-cancerogen* or ((cancer* or tumor* or tumour*or neoplas*) NEAR/3 treat*)):ti,ab,kw 121847

#5 [mh "Antineoplastic Agents"] OR [mh "Combined Modality Therapy"] OR [mh "Antineoplastic Combined Chemotherapy Protocols"] OR [mh "chemotherapy, adjuvant"] OR [mh "Antineoplastic Protocols"] OR [mh "Chemoradiotherapy"] OR [mh ^Radioimmunotherapy] 52004

#6 (Radio-therap* or radiotherap* or radiationtherap* or chemoradiotherap* or radiochemotherap* or protontherap* or radiosurg* or radio-surg* or irradiation* or x-ray-therap* or (therap* NEXT radiolog*) or IMRT? or IORT? or radioimmunotherap* or radio-immuno-therap* or ((radiat* or irradiat* or radioisotope* or radio-isotope* or chemoradio or chemo-radio or radiochemo or radio-chemo or proton or x-ray or xray) NEAR/2 (therap* or oncolog* or brachytherap* or brachy-therap*))):ti,ab,kw 52322

#7 [mh Radiotherapy] OR [mh "Radiotherapy, Adjuvant"] 10414

#8 ((((biologic* NEXT response NEXT modifier*) or BRM? or immune or imune or immunogenic or immunoglobulin or immune-globulin or immunological or immunomodul* or immune-modul* or immunomodur*) NEAR/2 (therap* or treatment or intervention or drug? or agent?)) or immunotherap* or imunotherap* or (immunity NEXT modulator*)):ti,ab,kw 19532

#9 [mh ^Immunotherapy] 2426

#10 #4 or #5 or #6 or #7 or #8 or #9 173354

#11 (AMH or (Anti-Mullerian NEXT Hormone*) or (Anti-Muellerian NEXT Hormone*) or (Antimullerian NEXT Hormone*) or (Antimuellerian NEXT Hormone*) or (Anti-Mullerian NEXT Factor*) oe (Anti-Muellerian NEXT Factor*) or (Mullerian NEXT Inhibiting NEXT Hormone*) or (Muellerian NEXT Inhibiting NEXT Hormone*) or (mullerian NEXT inhibitor*) or (muellerian NEXT inhibitor*) or testis or testes or testicle? or spermatogenes* or sperm* or semen or gametogenes*):ti,ab,kw 10931

#12 [mh Fertility] OR [mh Infertility] OR [mh Gonads] OR [mh ^"menopause, premature"] OR [mh ^Reproduction] OR [mh ^Gametogenesis] OR [mh ^Spermatozoa] 7029

#13 #11 or #12 15407

#14 #3 and #10 and #13 with Cochrane Library publication date Between Jan 2000 and Feb 2024 180

1. Borissov N, Haas Q, Minder B, et al. Reducing systematic review burden using Deduklick: a novel, automated, reliable, and explainable deduplication algorithm to foster medical research. Syst Rev. 2022;11(1):172. Published 2022 Aug 17. doi:10.1186/s13643-022-02045-9 [↑](#footnote-ref-1)
2. Bramer WM, Giustini D, de Jonge GB, Holland L, Bekhuis T. De-duplication of database search results for systematic reviews in EndNote. J Med Libr Assoc. 2016 Jul;104(3):240-3. doi: 10.3163/1536-5050.104.3.014. Erratum in: J Med Libr Assoc. 2017 Jan;105(1):111. doi: 10.5195/jmla.2017.128. [↑](#footnote-ref-2)
